# Residência Médica no Brasil na Era das Doenças Crônicas: A Necessidade da Residência em Medicina Cardiometabólica

**DOI:** 10.36660/abc.20210339

**Published:** 2022-03-10

**Authors:** Eduardo Thadeu de Oliveira Correia, Letícia Mara dos Santos Barbetta, Mayara Gabriele Toledo, Evandro Tinoco Mesquita, Jeffrey I. Mechanick

**Affiliations:** 1 Universidade Federal Fluminense Niterói RJ Brasil Universidade Federal Fluminense, Niterói, Rio de Janeiro, RJ – Brasil; 2 Instituto Cardiovascular Complexo Hospitalar de Niterói Niterói RJ Brasil Instituto Cardiovascular do Complexo Hospitalar de Niterói, Niterói, RJ – Brasil; 3 Centro de Educação e Treinamento Edson Bueno UnitedHealth Group Niterói RJ Brasil Centro de Educação e Treinamento Edson Bueno - UnitedHealth Group, Niterói, RJ – Brasil; 4 Icahn School of Medicine at Mount Sinai Divisions of Cardiology and Endocrinology, Diabetes and Bone Disease New York EUA Icahn School of Medicine at Mount Sinai – Divisions of Cardiology and Endocrinology, Diabetes and Bone Disease, New York – EUA

**Keywords:** Diabetes Mellitus, Obesidade, Hipertensão, Internato e Residência

## Introdução

Doenças cardiovasculares (DCV) são a principal causa de morte no Brasil, diminuindo significativamente a expectativa de vida, prejudicando a qualidade de vida, e afetando grandemente no Sistema de saúde unificado brasileiro. Entretanto, é importante destacar o enorme avanço na pesquisa cardiovascular e na cardiologia brasileira nas últimas 6 décadas, que contribuíram para aumentar a expectativa de vida de 54,1 anos em 1960 para 75,6 anos em 2018.^1^

**Figura 1:**
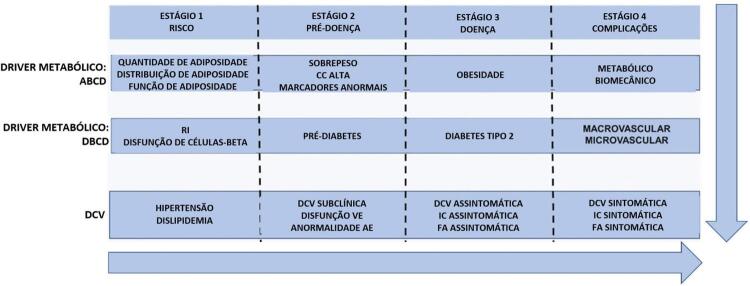
Doença crônica cardiometabólica: drivers metabólicos e etapas. Adaptado de (2). FA: fibrilação atrial; DCC: doença cardíaca coronária; IC: insuficiência cardíaca; RI: resistência à insulina; AE: atrial esquerda; VE: ventricular esquerda; CC: circunferência da cintura.

Apesar de, no último século, a cardiologia ter travado uma grande batalha contra as doenças agudas, tais como a endocardite e o infarto agudo do miocárdio, a pesquisa médica fez progressos inovadores no controle dessas doenças. Contudo, ao mesmo tempo, a era das doenças crônicas surgiu, em que os drivers principais (por exemplo, genética, ambiente, comportamento), os drivers metabólicos (por exemplo, obesidade, diabetes, colesterol alto e hipertensão), as comorbidades (por exemplo doença hepática gordurosa não alcoólica e doença renal crônica [DRC]), e os desfechos clínicos (por exemplo, doença cardíaca coronária, insuficiência cardíaca e fibrilação atrial) foram modelados para melhorar os resultados para o pacientes. Em um esforço para melhorar esse conceito, Mechanick et al.^2 , 3^ apresentaram o modelo de Doença crônica cardiometabólica (CMBCD), com foco no impacto de drivers primários e metabólicos no desenvolvimento de DCV, identificando as principais metas para reduzir a progressão de risco (CMBCD de estágio 1) para pré-doença (CMBCD de estágio 2), doença (CMBCD de estágio 3) e complicações (CMBCD de estágio 4). Essa classificação é mostrada na Figura 1 e marca uma nova era no cuidado de doenças cardiometabólicas.

Essa é uma estrutura cardiometabólica nova, combinada com a necessidade da participação do médico em um modelo de cuidado de pacientes com doenças cardiometabólicas em equipe, composta de médico especialista, nutricionista, educador físico, fisioterapeuta, psicólogo. Além disso, novas modalidades de estilo de vida estruturado, novas farmacoterapias e tecnologias exigem o treinamento de uma nova geração de médicos especialistas em medicina cardiometabólica (MC). Nesse trabalho, o argumento por trás da criação de uma residência em MC no Brasil será detalhado, e as propostas preliminares serão aprovadas em discussões futuras

### O caso atual do treinamento em cardiologia no Brasil e oportunidades de melhoria

Uma residência em cardiologia no Brasil geralmente dura 2 anos, durante os quais os residentes ganham experiência em procedimentos comuns no cenário do cuidado de pacientes ambulatoriais ou internados, com a opção de seguir fazendo uma super-residência e mais especializações. Entretanto, há melhorias importantes a serem feitas, com base na crescente prevalência de doenças cardiometabólicas, no desconhecimento dos fatores de risco, na proporção significativa dos pacientes não tratados adequadamente, no aumento do impacto de fatores de risco da carga das DCV não tratados adequadamente, e em uma infraestrutura de saúde ainda não preparada para lidar com esse problema.

### Roteiros de sucesso possíveis

**Figura 2:**
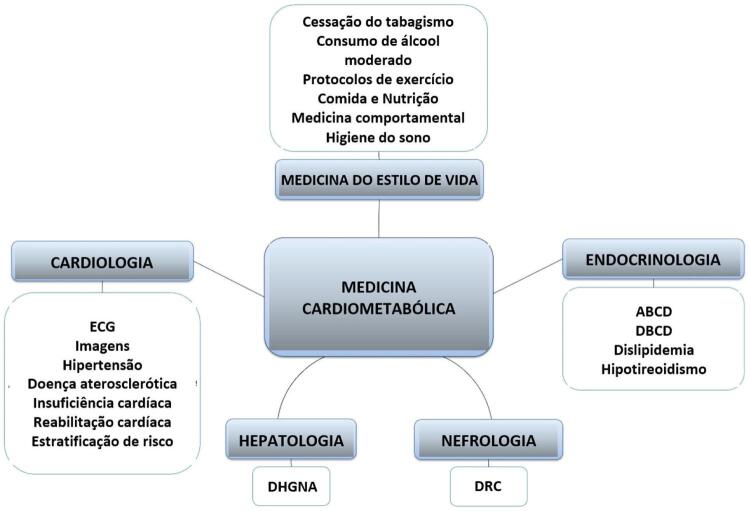
Principais componentes de uma residência médica em medicina cardiometabólica. ABCD: doença crônica baseada em adiposidade; DRC: doença renal crônica; DBCD: Doença crônica baseada em disglicemia; ECG: eletrocardiograma; DHGNA: doença hepática gordurosa não alcoólica.

**Figura 3:**
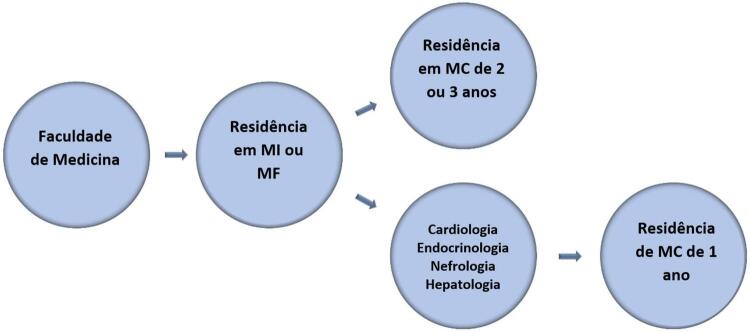
Possíveis roteiros de treinamento de uma residência em medicina cardiometabólica no Brasil. MC: medicina cardiometabólica; MF: medicina de família; MI: medicina interna.

Vários especialistas defendem a criação de uma residência em MC, o que daria aos médicos uma formação sólida para realizar o diagnóstico e tratamento de doenças crônicas, com foco em assuntos de cardiologia, endocrinologia, hepatologia, nefrologia, e medicina do estilo de vida.^4 - 7^ Isso ajudaria os médicos a oferecer um cuidado abrangente aos pacientes portadores de vários fatores de risco e doenças, reduzindo a fragmentação em várias especialidades, o que pode comprometer, atrasar e aumentar o custo do cuidado. Muitos artigos propuseram várias estruturas para programas de residência em Medicina Cardiometabólica (MC). Soroosh et al.^4^ propuseram três roteiros. Primeiro, haveria uma estrutura de treinamento composta de 2 a 3 anos de residência primária em MC depois da residência em medicina interna, durante a qual seriam cobertos tópicos aprofundados de cardiologia e endocrinologia. O componente de cardiologia incluiria eletrocardiografia, abordagens à hipertensão, prevenção de doença aterosclerótica (DA), procedimentos de medicina vascular, e outros métodos de imagem cardiovascular.^4^ O componente de endocrinologia consistiria no cuidado abrangente da obesidade e do diabetes orientado por diretrizes, bem como na gestão da síndrome metabólica (SM), distúrbios do metabolismo de lipoproteínas, hipogonadismo masculino e doenças da tireoide.^4^ Esse programa incluiria treinamento formal estruturado, incluindo cessação do tabagismo, higiene do sono, medicina comportamental e redução do stress, fisiologia do exercício, gestão do consumo excessivo de álcool, e envolvimento na comunidade.^4^ O segundo roteiro proposto por Soroosh et al.^4^ seria um programa de treinamento cardiometabólico de um ano após a residência de medicina interna (MI), endocrinologia, cardiologia ou nefrologia. Nessa opção, os primeiros 6 meses dessa super-residência estariam focados em tópicos cardiometabólicos centrais, e os próximos 6 meses seriam personalizados pelo residente. Por último, o terceiro roteiro abordado por Soroosh et al.^4^ seria um treinamento concomitante em MC com cursos de duração variável durante o tempo alocado para um programa de treinamento formal em cardiologia, endocrinologia, etc., com a vantagem de que qualquer médico poderia se especializar em MC, mas não seria um modelo tão aprofundado quando os demais. Em outra abordagem, McCarthy et al.^5^ propõem um programa de treinamento de um ano em MC, disponibilizado como residência para clínicos, tais como cardiologistas, endocrinologistas e nefrologistas. O residente faria rodízios em ambulatórios de cardiologia, endocrinologia e nefrologia além de passar um mês em ambulatórios de cardiologia feminina, medicina vascular e do sono, e controle do peso. Durante o ano, seriam cobertos tópicos relacionados a mudanças de estilo de vida, reabilitação cardíaca e abordagens nutricionais, diabetes, tratamentos antihiperglicemiantes e hipolipêmicos, escore de cálcio coronário, tomografia computadorizada cardíaca, abordagem à hipertensão, DRC, e fisiologia do exercício. Além disso, Eckel et al.6 propuseram, em outro trabalho, um programa de residência em MC de três anos, após a residência em MI, cobrindo tópicos em endocrinologia e cardiologia. Nesse modelo, seriam cobertos tópicos de obesidade, SM, diabetes e distúrbios do metabolismo de lipoproteínas, além da farmacologia dessas doenças. Em relação a tópicos de cardiologia, o foco seria na prevenção primária e secundária de DA, reabilitação cardíaca, interpretação de ecocardiograma e eletrocardiograma, e estratificação de riscos, bem como uma abordagem forte da hipertensão associada à medicina vascular. Além disso, durante esse treinamento, assuntos de medicina do estilo de vida seriam tratados constantemente. Finalmente, Reiter-Brennan et al.^7^ em outra publicação, propuseram um programa de treinamento amplo, após 2 ou 3 anos de prática médica, com um aprofundamento em endocrinologia e cardiologia, além de tópicos tais como bioestatística, epidemiologia, e psicologia comportamental. O principal pilar da parte cardiovascular seria o cuidado relacionado a DA, incluindo prevenção primária e secundária, fatores de risco, métodos de estratificação de risco, e escore de cálcio coronário. A reabilitação cardíaca também seria abordada. A parte endócrina cobriria diabetes, controle da hipertensão, SM, obesidade e distúrbios do metabolismo de lipoproteínas. Além desses, outro pilar desse treinamento seria uma abordagem forte a mudanças em estilo de vida, com ênfase na fisiologia da nutrição e exercício. As competências centrais a serem cobertas em residências de MC são representadas na Figura 2 . Considerando as propostas sugeridas por esses autores e considerando as disparidades e especificidades da população brasileira e da formação médica, em nossa opinião, duas propostas são mais promissoras para se criar uma residência em medicina cardiometabólica no Brasil. Uma primeira abordagem seria instituir um treinamento de MC após MI ou medicina de família (MF). Outra possibilidade seria estabelecer um programa de residência de um ano após terminar residência em cardiologia, endocrinologia, nefrologia ou hepatologia. Esses dois roteiros estão ilustrados na Figura 3 .

## Conclusões

Devido a um aumento significativo na prevalência e na incidência de doenças cardiometabólicas, as Sociedades Brasileiras de MI, MF, Cardiologia, Endocrinologia, Hepatologia e Nefrologia precisam iniciar uma discussão detalhada voltada para a criação de uma proposta formal para uma residência médica em MC a ser discutida no Conselho Federal de Medicina e apresentada na Comissão Nacional de Residência Médica para aprovação. Dessa forma, pode-se oferecer treinamento adequado a uma nova classe de médicos que vai unificar o cuidado de várias doenças crônicas em uma única especialidade, estimulando a pesquisa nessa área e reduzindo o risco de desenvolvimento de DCV.
