# Supplementary material for: Residência Médica no Brasil na Era das Doenças Crônicas: A Necessidade da Residência em Medicina Cardiometabólica
Source: Arq Bras Cardiol. 2022 Mar 10;118(3):655–8. [Article in Portuguese] doi: 10.36660/abc.20210339 (PMC8959041; doi:10.36660/abc.20210339)
Supplement: Translation

# Translation: Medical Residency in Brazil in the Era of Chronic Diseases: The Need for Cardiometabolic Medicine Residency

**Authors:** Eduardo Thadeu de Oliveira Correia, Letícia Mara dos Santos Barbetta, Mayara Gabriele Toledo, Evandro Tinoco Mesquita, Jeffrey I. Mechanick

**Affiliations:** 1 Brazil Universidade Federal Fluminense, Niterói, Rio de Janeiro, RJ – Brazil; 2 Brazil Instituto Cardiovascular do Complexo Hospitalar de Niterói, Niterói, RJ – Brazil; 3 Brazil Centro de Educação e Treinamento Edson Bueno - UnitedHealth Group, Rio de janeiro, RJ – Brazil; 4 USA Icahn School of Medicine at Mount Sinai – Divisions of Cardiology and Endocrinology, Diabetes and Bone Disease, New York – USA

**Keywords:** Diabetes Mellitus, Obesity, Hypertension, Internship and Residency

## Introduction

Cardiovascular diseases (CVD) are the leading cause of death in Brazil, significantly decreasing life expectancy, impairing quality of life, and causing a great impact on the Brazilian Unified Health System. However, it is important to highlight the enormous progress in cardiovascular research and in Brazilian cardiology over the past 6 decades, which have contributed to the increase in life expectancy from 54.^1^ years in 1960 to 75.6 years in 2018.^1^

Whereas in the last century, cardiology waged a major battle against acute diseases, such as endocarditis and acute myocardial infarction, medical research has made breakthrough advancements in the management of these conditions. Nonetheless, at the same time, the era of chronic diseases emerged, in which primary drivers (e.g., genetics, environment, and behavior), metabolic drivers (e.g., obesity, diabetes, high cholesterol, and hypertension), co-morbidities (e.g., non-alcoholic fatty liver disease and chronic kidney disease [CKD]), and clinical endpoints (e.g., coronary heart disease, heart failure, and atrial fibrillation) were modeled to improve patient outcomes. As an effort to improve this concept, Mechanick et al.^2 , 3^ introduced the Cardiometabolic-Based Chronic Disease (CMBCD) model, which focused on the impact of primary and metabolic drivers on the development of CVD, identifying key targets to reduce progression from risk (Stage 1 CMBCD) to pre-disease (Stage 2 CMBCD), disease (Stage 3 CMBCD), and complications (Stage 4 CMBCD). This classification is depicted in Figure 1 and marks a new era in the care of cardiometabolic diseases.

**Figure 1:** Cardiometabolic-Based Chronic Disease: metabolic drivers and stages. Adapted from (2). AF: atrial fibrillation; CHD: coronary heart disease; HF: heart failure; IR: insulin resistance; LA: left atrial; LV: left ventricular; WC: waist-circumference.

This novel cardiometabolic framework, coupled with the need for physician participation in a team care model of patients with cardiometabolic diseases, comprised of a specialist physician, nutritionist, physical educator, physical therapist, psychologist, as well as new structured lifestyle modalities, pharmacotherapies, and technologies, mandates the training of a new generation of cardiometabolic medicine (CM) physicians. In this paper, the rationale behind the creation of a CM fellowship in Brazil will be detailed, with preliminary proposals to be vetted in future discussions.

### The present case of brazilian cardiology training and opportunities for improvement

A cardiology fellowship in Brazil typically lasts for 2 years, during which fellows obtain experience in common procedures in both outpatient and inpatient care settings, with the option to pursue subsequent super-fellowships and further specialization. However, there are key improvements to be made, based on the increasing prevalence of cardiometabolic diseases, risk factor unawareness, significant proportion of patients not adequately treated, increased impact of inadequately treated risk factors on the burden of CVD, and a healthcare infrastructure not yet poised to address this problem.

### Possible Pathways to Success

Many experts have advocated the introduction of a CM fellowship, which would provide physicians a solid background for diagnosis and treatment of chronic diseases, focusing on topics of cardiology, endocrinology, hepatology, nephrology, and lifestyle medicine.^4 - 7^ This would help physicians provide comprehensive care to patients with multiple risk factors and conditions, reducing fragmentation into different specialties, which can compromise, delay, and increase the cost of care. Several articles have proposed various structures for CM fellowship programs. Soroosh et al.^4^ proposed three pathways. First, there would be a training structure consisting of 2 to 3 years of primary fellowship in CM after internal medicine (IM) residency, during which in-depth topics in cardiology and endocrinology would be covered. The cardiology component would include electrocardiography, approaches to hypertension, prevention of atherosclerotic disease (AD), vascular medicine procedures, and other cardiovascular imaging methods.^4^ The endocrinology component would consist of guideline-directed comprehensive diabetes and obesity care, as well as the management of metabolic syndrome (MS), lipoprotein disorders, male hypogonadism, and thyroid diseases.^4^ This program would include a formal structured training, including tobacco cessation, sleep hygiene, behavioral medicine and stress reduction, exercise physiology, management of alcohol abuse disorder, and community engagement.^4^ The second pathway proposed by Soroosh et al.^4^ would be a 1-year cardiometabolic training program after IM, endocrinology, cardiology, or nephrology fellowship. In this option, the first 6 months of this super-fellowship would be focused on core cardiometabolic topics, and the next 6 months would be customized by the fellow. Finally, the third pathway approached by Soroosh et al.^4^ would be concurrent training in CM with courses of varying length during time allocated to a formal training program in cardiology, endocrinology, etc., with the advantage that any physician would be able to specialize in CM, but it would not be as in-depth as the other models. In another approach, McCarthy et al.^5^ proposes a 1-year training program in CM, available as a fellowship for clinicians, such as cardiologists, endocrinologists, and nephrologists. The fellow would undergo rotations in cardiology, endocrinology, and nephrology outpatient clinics, in addition to 1 month in the women’s cardiology, vascular and sleep medicine, and weight management outpatient clinics. Throughout the year, topics related to lifestyle changes, cardiac rehabilitation and nutritional approaches, diabetes, antihyperglycemic and hypolipemic therapies, coronary artery calcium quantification, cardiac computerized tomography, approach to hypertension, CKD, and exercise physiology would be covered. Beyond that, Eckel et al.,^6^ in another paper, proposed a 3-year fellowship program in CM, after IM residency, covering topics in endocrinology and cardiology. In this model, the topics of obesity, MS, diabetes and lipoprotein disorders, and the pharmacological therapies of these conditions would be covered. Regarding cardiology topics, the focus would be on primary and secondary prevention of AD, cardiac rehabilitation, interpretation of echocardiogram and electrocardiogram, and risk stratification, as well as a strong approach to hypertension associated with vascular medicine. Moreover, during this training, lifestyle medicine subjects would be constantly addressed. Finally, Reiter-Brennan et al.,^7^ in another publication, proposed a broad training program, after 2 or 3 years of medical practice, with a more in-depth study of endocrinology and cardiology, plus such topics as biostatistics, epidemiology, and behavioral psychology. The main pillar of the cardiovascular section would be the care related to AD, including primary and secondary prevention, risk factors, methods of risk stratification and quantification of artery calcification; cardiac rehabilitation would also be covered. In the endocrine section, diabetes, hypertension management, MS, obesity, and lipoprotein disorders would be covered. In addition to these, another pillar of this training would be a strong approach to lifestyle changes, with emphasis on the physiology of nutrition and exercise. Core competencies that should be covered in CM residencies and fellowships are covered in Figure 2 .

**Figure 2:** Core components of cardiometabolic medicine fellowship. ABCD: Adiposity-Based Chronic Disease; CKD: chronic kidney disease; DBCD: Dysglycemia-Based Chronic Disease; ECG: electrocardiogram; NAFLD: non-alcoholic fatty liver disease.

Given the proposals suggested by these authors and considering the disparities and specificities of the Brazilian population and medical training, in our view, two proposals are most promising to establish a cardiometabolic fellowship in Brazil. A first approach would be to institute CM training after IM or Family Medicine (FM). Another way would be to establish a 1-year fellowship program after completing cardiology, endocrinology, nephrology, or hepatology fellowships. These two pathways are illustrated in Figure 3 .

**Figure 3:** Possible training pathways of a cardiometabolic medicine fellowship in Brazil. CM: cardiometabolic medicine; FM: family medicine; IM: internal medicine.

## Conclusions

Due to the significant increase in the prevalence and incidence of cardiometabolic diseases, the Brazilian Societies of IM, FM, Cardiology, Endocrinology, Hepatology, and Nephrology must begin a thorough discussion centered on the creation of a formal proposal of a medical fellowship in CM to be discussed at the Brazilian Medical Association and presented in the National Medical Residency Commission for approval. In that way, proper training can be offered to a new class of physicians that will unify the care of multiple chronic diseases under one specialty, stimulating research in this area and reducing the risk of CVD development.
